# Note from the editors: Wave goodbye and say hello - *Eurosurveillance* to launch new website soon

**DOI:** 10.2807/1560-7917.ES.2017.22.38.30617

**Published:** 2017-09-21

**Authors:** Eurosurveillance editorial team

**Affiliations:** 1 European Centre for Disease Prevention and Control, Stockholm, Sweden

It is time to say good bye to the static old *Eurosurveillance* website that served its purpose over the course of many years. In a reader survey, several years ago, respondents mentioned as advantages of the site that it had an easy navigation structure, focussed on content and that it was robust with good response times. Respondents also flagged that while they were overall happy with *Eurosurveillance* and its content, they missed some important features that several other journals offered.

We have been developing a new website that, while preserving the advantages of the old site, will respond better to our readers’ needs and interests. This week will be the last time that we will send the Table of Contents (TOC) containing links to articles published on the old website and we are glad to announce the imminent launch of a new website in the afternoon of Monday 25 September.

The ‘new’ *Eurosurveillance* will offer social bookmarking, a more refined search, article-level metrics, a citation export feature and the opportunity to download PowerPoints for figures and tables for all users. In addition, everyone who registers via the new website will be able to set up and save content searches and specific alerts, including citation alerts and saved search alerts, so that authors can be automatically notified when their work is cited or users receive an alert when a paper covering a topic of particular interest to them is published. These are but a few of the new, useful features.

The new website will remain both open access and free of article processing charges.

It is important to note that in order to follow European Union data protection rules, current subscribers to the weekly TOC will automatically be unsubscribed from *Eurosurveillance* and all collected personal data will be destroyed. Everyone who wishes to benefit from the new features and to receive the weekly TOC is invited to say hello to the new website and visit it at www.eurosurveillance.org to register and subscribe once it is launched.

We look forward to receiving your feedback (eurosurveillance@ecdc.europa.eu) and hope our readers and authors will like the new *Eurosurveillance*.

